# Fetal Lipoblastoma Presenting as a Retroperitoneal Tumor in Adults: A Case Report

**DOI:** 10.7759/cureus.48075

**Published:** 2023-10-31

**Authors:** Frida L Sanchez, Guadalupe Olvera, Ricardo J Arreola Peralta, Cesar R Barrera

**Affiliations:** 1 Surgery, Hospital General Regional 1, Morelia, MEX; 2 General Surgery, Hospital General Regional 1, Morelia, MEX; 3 Colorectal Surgery, Hospital General Regional 1, Morelia, MEX

**Keywords:** nonspecific abdominal pain, retroperitoneal tumor, explorative laparotomy, retroperitoneal lipoblastoma, retroperitoneal, laparotomy, abdominal tumor, abdominal pain, plag1 oncogene, lipoblastomatosis

## Abstract

Lipoblastoma (LB) is a rare benign mesenchymal soft tissue neoplasm that most frequently occurs in childhood. In this case, we describe a 33-year-old female with intermittent abdominal pain due to the presence of a slowly growing abdominal tumor. We will explain the ideal diagnostic protocol and compare it with the diagnostic approach performed at a second level of care with limited resources. A simple abdominal tomography confirmed the presence of the retroperitoneal tumor, which led to the planning of surgical management for these tumor cases. A "complete resection" is considered the ideal approach, along with the follow-up to rule out any complications.

## Introduction

Lipoblastoma (LB) is a rare benign mesenchymal soft tissue neoplasm that occurs most frequently in childhood and rarely in adults [1,2]. It has a prevalence of 0.6% of benign soft tissue tumors [3]. The vast majority is detected in children under three years old, with more than 80% of cases before three years old and 40% before one year old [1,3]. It is more common in males than in females [1,2,4]. It is a neoplasm of embryonic white blood cells, which usually presents as a well-defined tumor or presents as a diffuse process known as localized lipoblastomatosis (LBS) [2]. Approximately 70% of these tumors occur in the extremities, trunk, head, and neck; however, retroperitoneal lipoblastomas are less common (<5% of cases) [1-3]. The differential diagnosis of the tumor is broad and includes sarcomas, neuroblastomas, and teratomas [1]. Although histologically it is benign, it has a locally invasive behavior with a risk of recurrence if it is not completely removed [5]. We report a case of a 33-year-old female with a diagnosis of fetal lipoblastoma after a histopathology report and how we did the diagnosis and management was realized.

## Case presentation

A 33-year-old female was referred from the family medicine unit for evaluation by the general surgery service due to the presence of a three-month-old left lumbar tumor accompanied by intermittent abdominal pain, periods of constipation alternating with diarrhea, nausea, and vomiting, without the presence of feverish peaks and channeling gases and with normal diuresis.

In the interrogation, her mother has a diagnosis of diabetes mellitus, and her father has a history of diabetes mellitus and systemic arterial hypertension. She denied any family history of cancer.

She stated that she did not suffer from chronic degenerative diseases; in her surgical history, she had a cesarean section in 2018 and a cesarean section and hysterectomy in 2021; she also denied allergies, transfusions, chronic medication intake, alcoholism, smoking, and drug addiction.

In the initial consultation in the general surgery department, a physical examination was performed, revealing an abdominal tumor in the lower left region, approximately 5×9 cm in size. The tumor was dull on percussion, had a soft consistency, was not very mobile, and caused pain upon mobilization. The rest of the abdomen showed no apparent abnormalities. Bilateral Giordano tests were negative, with no pain in ureteral points. The patient had intact extremities and preserved muscle strength (scored 5/5 on the Daniels scale), and pulses were present with immediate capillary refill. Complementary studies available within the hospital unit were requested for reevaluation during the second consultation.

**Figure 1:**
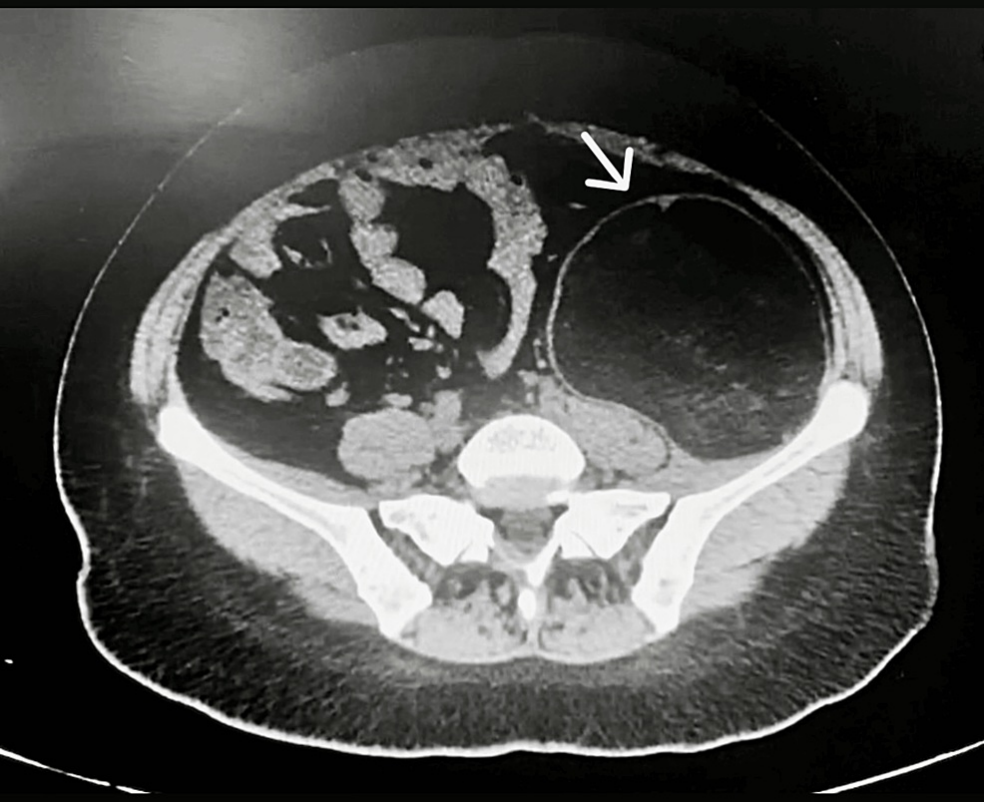
Simple abdominal tomography in axial section A heterogeneous retroperitoneal mass with well-defined edges is observed, which causes a significant mass effect in intra-abdominal organs

**Figure 2:**
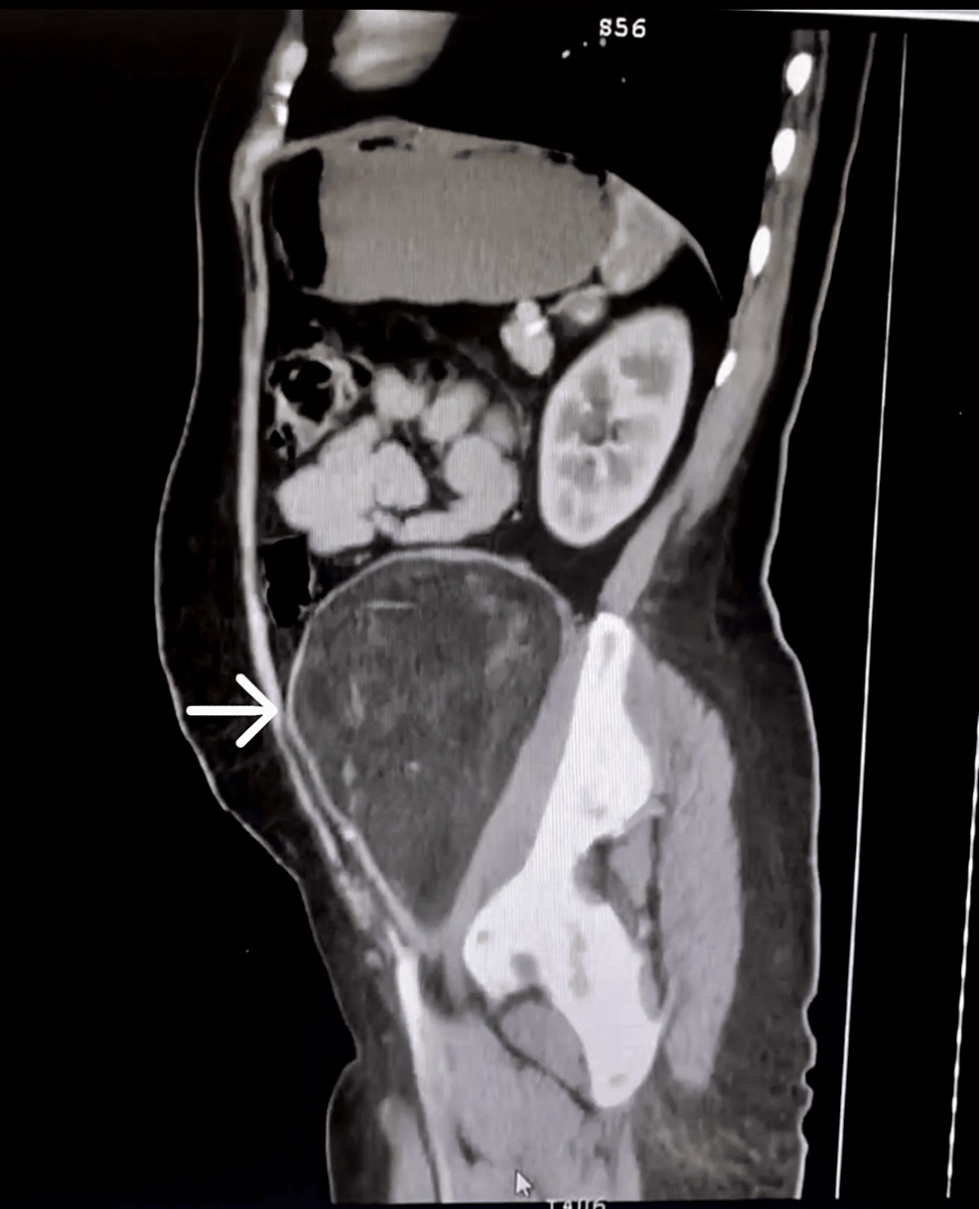
Simple abdominal tomography in sagittal section

**Figure 3:**
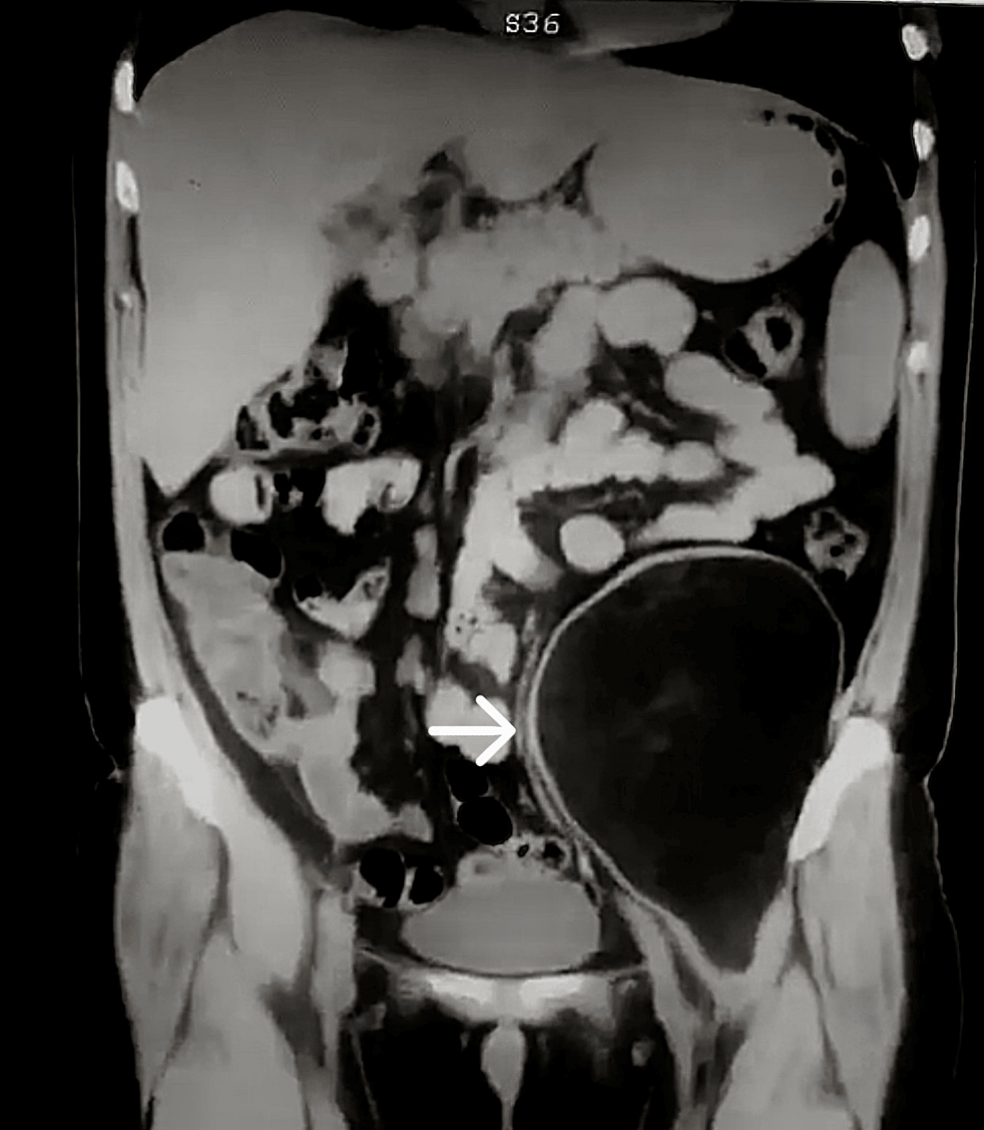
Simple abdominal tomography in coronal section

A study protocol was carried out, considering the resources of the hospital. Laboratory studies reported normal results, including tumor markers: alpha-fetoprotein (AFP), 1.32 UI/mL; carcinoembryonic antigen (CEA), 0.487 ng/mL; cancer antigen (CA) 125, 7.93 U/mL; and CA 19-9, 3.97 U/mL. A simple abdominal tomography was performed in axial (Figure 1), sagittal (Figure 2), and coronal (Figure 3) sections, revealing a left retroperitoneal tumor extending into the pelvic cavity. It appeared well-delimited, heterogeneous, and without apparent involvement of adjacent structures and caused the displacement of neighboring organs (the small and large intestine) (Figures 1-3). At that time, magnetic resonance imaging (MRI) or contrast-enhanced CT scans were not available in the unit.

In the second assessment, the patient commented that she wanted elective surgery to be scheduled as soon as possible for fear of malignancy. She was discussed taking a biopsy before definitive management according to protocol, which she did not accept, and was scheduled for elective "exploratory laparotomy" surgery for complete resection.

**Figure 4:**
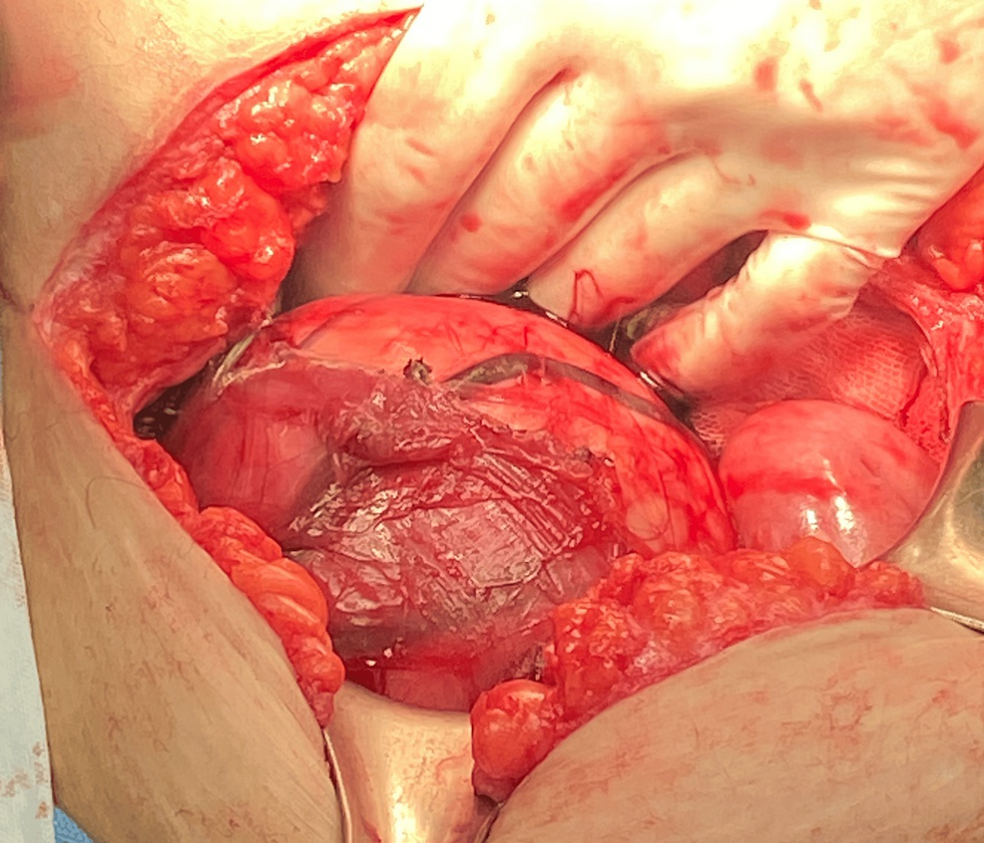
Tumor exposure to the opening of the abdominal cavity along the midline Tumor with extension to the pelvic cavity, highly vascularized, with friable tissue

**Figure 5:**
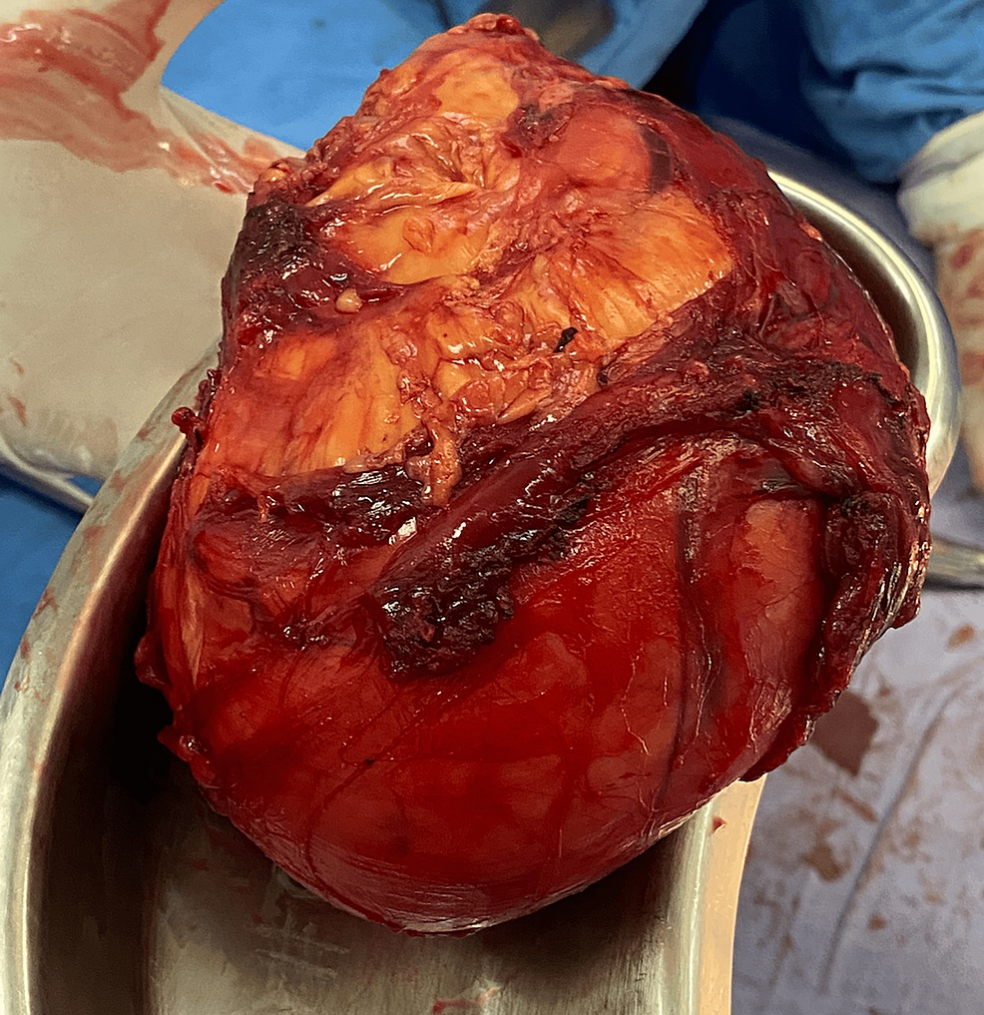
Retroperitoneal tumor Complete remotion of the tumor, which was a solid, highly vascularized retroperitoneal tumor

An incision was made in the infraumbilical midline, with the exposure of the tumor (Figure 4), and a complete resection was performed with transoperative findings reporting "tumor located in the left retroperitoneal region, approximately 15 cm in vascularized diameter, with a capsule that infiltrates psoas muscle tissue. Complete resection was performed without injury to adjacent structures and partial resection of the psoas muscle" (Figure 5). The piece was sent to the pathology service.

The piece was sent to the pathology service for the definitive histopathological report.

The patient was admitted to the hospitalization floor where the enhanced recovery after surgery (ERAS) protocol was performed. She was started on a liquid diet four hours after surgery without complications; the urinary catheter was removed, and she began walking six hours later, and intravenous fluids were subsequently reduced. Nonsteroidal anti-inflammatory drugs (NSAIDs) were reduced, and a soft diet was progressed on the first postsurgical day. She did not experience nausea or vomiting, so she was discharged without complications on the second postoperative day. She had a follow-up appointment in an outpatient clinic one month after surgery: asymptomatic with adequate healing.

**Figure 6:**
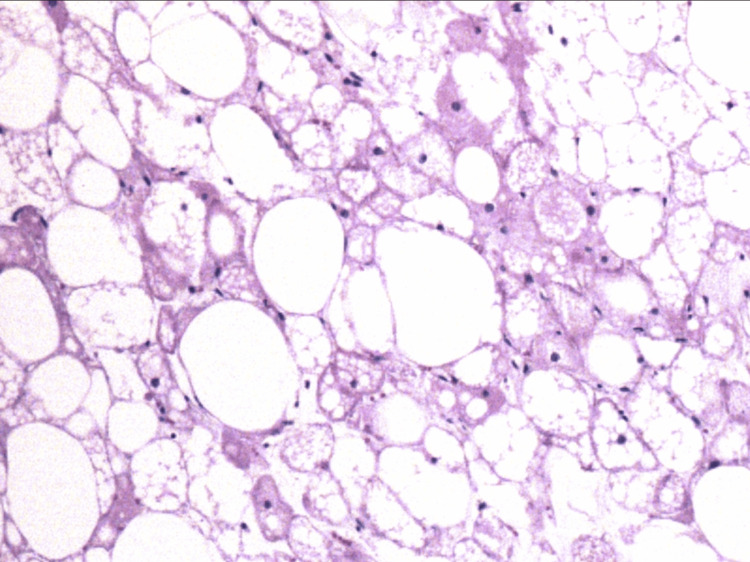
Histological sections stained with hematoxylin-eosin Mesenchymal neoplasia is observed and characterized by adipocytes in different stages of maturation and of different sizes; some are univacuolated, large with nuclei displaced to the periphery, round or oval with hyperchromasia, and alternating with a smaller number of multivacuolate adipocytes and central nucleus

**Figure 7:**
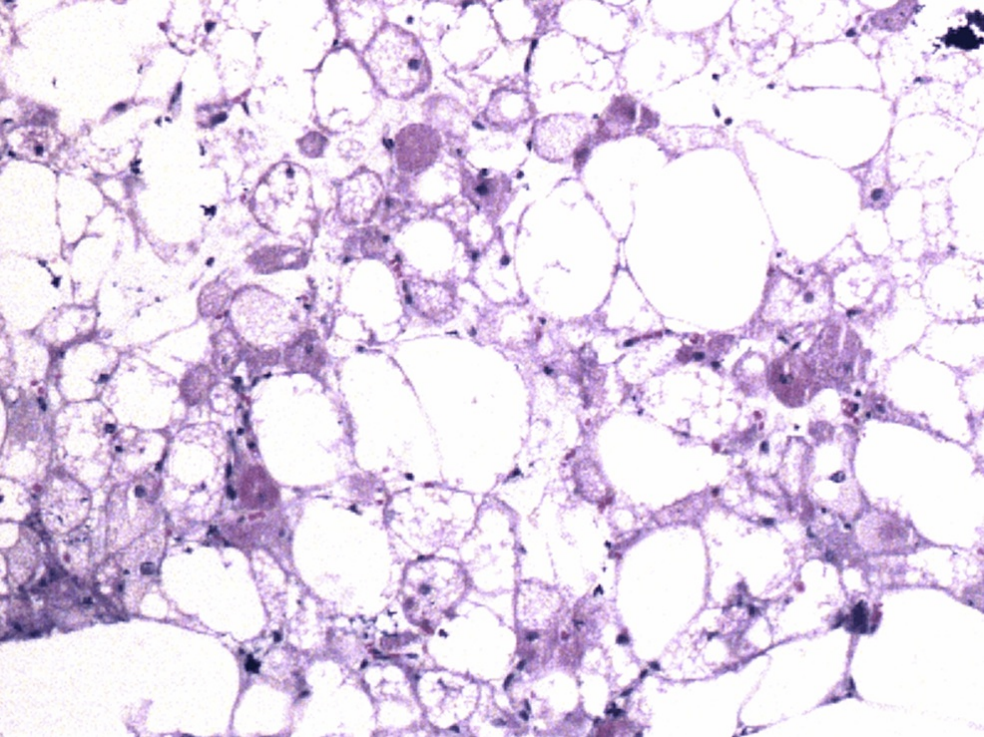
Histological sections stained with hematoxylin-eosin

Pathological anatomy reported macroscopically "ovoid specimen measuring 14×12.5×8 cm partially covered by a thin membrane, which has a solid surface and was discreetly lobed and light yellow with multifocal yellowish areas." On histopathological examination, it was found to be compatible with fetal lipoma (benign lipoblastoma) (Figures 6, 7).

Subsequent reassessment after six months without data was suggestive of recurrence. The laboratory result reported was normal, and physical examination showed adequate healing process without palpable tumors.

## Discussion

Epidemiologically, lipomatous tumors represent 6% of soft tissue neoplasms in children, representing up to 30% in some reports [4,6]. The majority of these tumors are 64%-90% lipomas, and the rest correspond to liposarcomas, 1%-4%; LB, 5%-30%; and hibernomas, 2% [4].

LB are soft tissue tumors composed of embryonic/fetal fat; they are characterized by a benign nature, early presentation, and fast growth [1]. They are diagnosed almost exclusively in the pediatric population, and the majority of cases, around 90%, are found in infants and children under three years of age [1,2]. They have a predilection for the male sex [7,8].

Two clinic pathological forms have been described: circumscribed and diffuse forms. The most common is the circumscribed one, located in superficial soft tissues. The diffuse form, also called LBS, originates in deep soft tissues and presents with an infiltrative pattern with a tendency to recurrence [7].

LB usually occurs in the extremities, trunk, head, and neck; other less frequent locations are the axilla, groin, lips, mediastinum, retroperitoneum, peritoneum, and carotid gland [3,6].

Its typical presentation is as a solitary soft palpable mass (47%), usually painless, or abdominal distention (29%) [9-11]. Most LB have a size of less than 5 cm [3].

The main diagnostic aids are ultrasound, tomography, and magnetic resonance [3]; in the plain radiograph, radiopaque images of the mass in soft tissues are observed in most cases, while on ultrasound, hyperechoic images with hypoechoic halos are seen [7]. On the other hand, magnetic resonance imaging (MRI) is considered the study with the greatest sensitivity [1,11], since it evaluates the location of the tumor and its size, composition, and adjacent organs and provides important information for surgical resection [3]. However, MRI cannot differentiate between different adipose tissue tumors, since there are no pathognomonic signs or lesions associated with LB [3].

Patients who present a retroperitoneal mass may benefit from performing a fine-needle aspiration puncture (FNAB) preoperatively to determine the extent of surgical management [5,12].

Resection and pathological examination offer a definitive diagnosis; currently, definitive diagnosis includes immunohistochemistry [1]. Early complications include bleeding, pain, infection, and injury to neighboring organs [12].

Morphologically, LB is mainly composed of primitive spindle mesenchymal cells and adipocytes in various stages of maturation. In particular, it contains lobes of mature adipocytes of different sizes divided by fibrous septa, with a fine capillary network and mucinous stroma [3].

From a histopathological perspective, one of the main differential diagnoses to consider is myxoid liposarcoma (ML). It is extraordinarily rare in children, composed of lipoblasts that may sometimes have a fusiform appearance, a myxoid matrix, well-vascularized septa, and a lobulated appearance, although less pronounced than in LB. The key distinction is the presence of cellular atypia, which is characteristic of liposarcoma but not seen in LB [5].

In actuality, immunohistochemistry gives the definitive diagnosis; it shows a simple pseudodiploid karyotype [3]. Currently, in cytogenetic studies, it has been associated with 8q11-13 and the PLAG1 oncogene in 82%, as opposed to lipomas in 3% [1,2,11]. This acts as a transcription regulator and is not expressed in adult tissue [3]. Other tumors caused by pleomorphic adenoma gene 1 (PLAG1) overexpression include pleomorphic adenoma of the salivary glands, hepatoblastoma, and acute myeloid leukemia [3]. Other markers are present in LB cluster of differentiation 34 (CD34), S-100, desmin, and p1, where positive S-100 and negative P16 are reported, while the expression of CD34 and desmin is usually observed in primitive mesenchymal cells [3].

The long-term prognosis of LB is usually excellent. The recurrence report rates range from 9% to 22%, which is attributed to the incomplete initial excision of the tumor [1].

In hospitals in developing countries, there is a shortage of resources, which can be detrimental to timely diagnosis and ideal management. In this case, no contrast study, biopsy, or immunohistochemistry was performed, which is considered within the diagnostic approach and appropriate surgical planning.

## Conclusions

In the study, a case of fetal lipoblastoma was presented, which is a neoplasm that occurs mostly in childhood and is considered uncommon in adults, thus being a diagnostic challenge for the general surgeon. Within the approach to abdominal pain, the presence of neoplasms (benign and malignant) must be considered as a cause to carry out an adequate diagnostic protocol considering the resources available in the hospital unit for confirmation and thus planning the surgical treatment to avoid complications and recurrence.
